# Factors Associated With Elevated C‐Reactive Protein Levels in Women With Systemic Lupus Erythematosus Followed at the Cocody University Hospital, Abidjan, Côte d’Ivoire

**DOI:** 10.1155/jimr/4741663

**Published:** 2026-11-04

**Authors:** Adjoumanvoulé Honoré Adou, Abidou Kawelé Coulibaly, Lasme Charline Roselle Memel, Kouabla Liliane Siransy

**Affiliations:** ^1^ Department of Fundamental and Bioclinical Sciences Félix Houphouët-Boigny University Abidjan Côte d’Ivoire univ-fhb.edu.ci; ^2^ Department of Medicine and Medical Specialties Félix Houphouët-Boigny University Abidjan Côte d’Ivoire univ-fhb.edu.ci; ^3^ Department of Fundamental and Bioclinical Sciences Alassane Ouattara University Bouaké Côte d’Ivoire univ-ao.edu.ci

**Keywords:** biomarkers, Côte d’Ivoire, C-reactive protein (CRP), hypercreatininemia, obesity, proteinuria, renal involvement, systemic inflammation, systemic lupus erythematosus (SLE)

## Abstract

Systemic lupus erythematosus (SLE) is a chronic autoimmune connective tissue disease characterised by multivisceral involvement and the presence of autoantibodies. In this context, C‐reactive protein (CRP) holds a particular position: a moderate rise accompanies autoimmune flares, whereas a sharp elevation suggests an infection or specific inflammatory manifestations. This study aimed to analyse the factors associated with increased CRP levels in women with lupus followed at the Cocody University Hospital in Abidjan. This was a cross‐sectional observational study including 62 adult women with SLE (ACR/EULAR criteria). Collected variables included socio‐demographic, clinical, biological and immunological data. CRP was categorised into three groups: (<6 mg/L, 6–20 mg/L and >20 mg/L). Univariate and multivariate analyses were performed using binary logistic regression, supplemented by the estimation of marginal effects. The results show a mean CRP concentration of 28.2 ± 21.2 mg/L. A CRP level >20 mg/L was common and significantly associated with renal manifestations, arthralgia/arthritis and the severity level of pain. Biologically, proteinuria ≥0.5 g/24 h, hypercreatininemia and leukocytosis were strongly correlated with elevated CRP. In multivariate regression, only hypercreatininemia remained significant (odds ratio [OR] = 24.8; *p* = 0.011). The estimation of marginal effects confirmed its major impact (+47.5 probability points), as well as that of proteinuria (+31.3 points) and obesity (+14.1 points), while overweight appeared protective (−19.2 points). The SLE Disease Activity Index (SLEDAI) score was positively but modestly correlated with CRP (+1.4 probability points/unit). The model’s ROC curve showed excellent performance (area under the ROC curve [AUC] = 0.84). In conclusion, elevated CRP levels were primarily associated with renal manifestations, notably proteinuria and elevated serum creatinine, as well as with disease activity. CRP could therefore serve as a complementary biomarker in the assessment of patients with SLE, particularly in the presence of renal involvement. However, these results should be interpreted as associations and require confirmation by longitudinal studies.

## 1. Introduction

Systemic lupus erythematosus (SLE) is a chronic, heterogeneous autoimmune connective tissue disease characterised by numerous visceral manifestations and the presence of autoantibodies [1]. The management of SLE relies on standardised clinical and laboratory monitoring and the judicious use of biomarkers to guide therapeutic decisions. The updated EULAR guidelines emphasise these principles and the need for decision‐aiding tools applicable in routine practice [2]. In addition to the autoantibodies that constitute its immunological signature, SLE is characterised, as the disease progresses, by the expression of numerous markers of inflammation, including C‐reactive protein (CRP). In SLE,CRP exhibits a distinctive pattern: it generally remains moderately elevated during isolated autoimmune flares, whereas a marked increase is more suggestive of a concurrent infection or certain inflammatory conditions such as vasculitis [3, 4]. In several hospital cohorts, CRP thresholds around 24 mg/L have been proposed as practical indicators of infection in febrile lupus patients, to be interpreted within the clinical context [5].

In sub‐Saharan Africa, and particularly in West Africa, the clinical presentation of SLE occurs in an environment with a high burden of infection and where access to specialist care remains unequal, which complicates the interpretation of elevated CRP levels. Epidemiological reviews show that infection and cardiovascular complications contribute significantly to excess mortality in SLE, whilst, in practice, the early differentiation between a flare‐up and an infection remains a major challenge for patient safety [6]. In Ghana, a prospective cohort study demonstrated the influence of the APOL1 genotype on kidney damage and prognosis, whilst in Nigeria, a recent multi‐centre study detailed the clinical and biological characteristics in hospital settings [7, 8]. In Abidjan, hospital‐based research highlights the burden of disease and the impaired quality of life among patients followed at the Cocody University Hospital, underscoring the value of simple tools for clinical decision‐making [9].

In malaria‐endemic areas, malaria often causes a significant increase in the CRP. This elevation can complicate the assessment of a febrile lupus patient presenting with elevated CRP. This reality reinforces the value of combined approaches (clinical, targeted microbiology, procalcitonin and simple ratios) to correctly interpret elevated CRP [10].

In this context, it is important to identify the factors contributing to elevated CRP levels in lupus patients, to distinguish between those related to infection, disease activity or other factors, and to draw conclusions regarding the implications for diagnosis and treatment adjustment. Our objective was therefore to analyse the factors that may be associated with elevated serum CRP levels in patients with SLE at the Cocody University Hospital.

## 2. Materials and Methods

This was a cross‐sectional analytical study based on clinical and laboratory data from patients with SLE. The medical records of patients hospitalised or seen in routine outpatient consultations at the Rheumatology Department of the Cocody University Hospital in Abidjan were systematically reviewed over a 4‐month period from March 2025 to June 2025.

The study included adult women (aged ≥18 years) with a confirmed diagnosis of SLE according to international criteria (ACR/EULAR) who had given their consent for the secondary use of their data. Excluded were all patients receiving treatment with corticosteroids and/or immunosuppressants prior to the biological assessment, as well as those whose records were incomplete and did not allow the primary data extraction, patients with an active infection at the time of the study, known liver disease, pregnancy, or another concomitant inflammatory or autoimmune disease. An exhaustive non‐probability sampling was carried out by consecutively including all records meeting the eligibility criteria during the study period, resulting in a total of 62 patients.

A single clinical and biological assessment point was retained for each woman, including the CRP measurement. Treatment information documented in the medical records was collected for descriptive purposes. The study did not include repeated CRP measurements before and after treatment.

Data collection was carried out using an anonymous questionnaire to ensure the confidentiality of the information. The variables included socio‐demographic characteristics (age and body mass index) and medical history. Clinical and disease‐related parameters included the SLE Disease Activity Index (SLEDAI) [11], pain assessment using a visual analogue scale (VAS) [12], the level of clinical severity, disease status (flare‐up or remission) and response to treatment.

CRP levels were measured by immunoturbidimetry on a Cobas c6000 automated analyser (Roche Diagnostics) using the Tina‐quant CRP IV (CRP4) reagent. This method, based on the agglutination of latex particles coated with anti‐CRP antibodies, offers a measurement range of 0.6–350 mg/L, a detection limit of 0.3 mg/L and a quantification limit of 0.6 mg/L. The recommended reference value is <5 mg/L, but each laboratory must verify its transferability. In the present study, a CRP level >6 mg/L was considered positive [13]. Routine laboratory data, including a complete blood count and tests for specific autoantibodies (antinuclear antibodies [ANAs], anti‐double‐stranded DNA antibodies, etc.), were included in the analysis [14].

### 2.1. Treatment of the Dependent Variable (Prior to the Statistical Analysis Section)

For statistical reasons, the dependent variable corresponding toCRP concentration was treated as a categorical variable. CRP levels were divided into three categories: <6 mg/L (normal), 6–20 mg/L (moderately elevated) [15, 16] and >20 mg/L (highly elevated) [5, 16, 17].

Participants were classified into two age groups: 18–45 years (period of high incidence corresponding to childbearing age) and > 45 years (beyond the peak period, including late‐onset forms). This categorisation is based on the epidemiology of SLE, which peaks between 15 and 45 years of age, and on reviews defining late‐onset forms as those occurring from the age of 50 onwards, as well as on local case series confirming the existence of cases in people over the age of 45 [6, 18–21].

### 2.2. Statistical Analysis

Quantitative variables were expressed as means ± standard deviation, and qualitative variables were expressed as percentages. Relationships between variables were explored using univariate and then multivariate analyses. For the multivariate analysis, a multinomial regression (CRP <5/5–20/>20 mg/L) was considered, but instabilities (near‐separation, limited sample sizes) led to dichotomising CRP at 20 mg/L (≥20 vs. <20 mg/L), a threshold validated in the literature [5, 17]. The outcome was therefore modelled using binary logistic regression on complete cases, with categorical variables coded using a reference category. The final model included age, BMI, proteinuria ≥0.5 g/24 h, elevated serum creatinine and SLEDAI score. Sensitivity analyses confirmed the robustness of the model, the results of which are presented as adjusted odds ratios (ORs) with 95% confidence intervals (CI). From this adjusted model, average marginal effects (AMEs) were calculated to estimate the average change in probability of CRP ≥ 20 mg/L associated with each factor. The model’s performance was assessed using the area under the ROC curve (AUC). All analyses were performed using SPSS Statistics v29 (IBM Corp.) and Python 3.11 (statsmodels 0.14.2). Correlation plots were generated using GraphPad Prism v10.2.0 (392). The significance threshold was set at *p* < 0.05.

### 2.3. Ethical Considerations

The study was carried out in accordance with the ethical principles set out in the Declaration of Helsinki. It was approved by the Ethics Committee of SIHIO‐TS (Ivorian Society of Haematology, Immunology, Oncology, and Blood Transfusion) under Reference Number 53_2025‐SIHIOTS was published on February 14, 2025. The secondary use of data was authorised by the participants’ informed consent, with complete anonymisation prior to the analysis.

## 3. Results

### 3.1. Basic Descriptive Characteristics

The study involved 62 women. Table 1 presents the main clinical, therapeutic and laboratory characteristics of the study population. The majority of patients reported no history of a first lupus flare‐up (83.9%). The articular form was the most common type of flare‐up (29.0%), although nearly half of the cases were not specified in the records (45.2%). Regarding nutritional status, 41.9% of patients had a normal BMI and 38.7% were overweight. Renal manifestations were observed in 32.3% of patients. The most common clinical presentations were cutaneous and mucosal lesions and joint pain (32.3%, respectively). In terms of treatment, synthetic antimalarials were used in 74.2% of patients, whilst immunosuppressants were prescribed in 19.4%. Clinical improvement was noted in 51.6% of patients. Proteinuria ≥0.5 g/24 h and hypercreatininaemia were observed in 37.1% and 32.3% of patients, respectively. Antiphospholipid syndrome was found in 9.7% of cases. ANAs had a median of 1185 (2.8–1280), whilst anti‐dsDNA and anti‐Sm had median values of 77 (8–600) and 19.5 (2–320), respectively. Anti‐SSA and anti‐SSB antibodies showed medians of 71.5 (1–640) and 12 (2–640) (Tables 1 and 2).

**Table 1:** Clinical parameters and treatment‐related parameters.

Parameters	Categories	*n*	%
History of first lupus flare	No	52	83.9
	Yes	10	16.1

History of flare type	Cutaneous	2	3.2
	Pulmo‐cardiac	2	3.2
	Articular	18	29.0
	Articular‐cutaneous	4	6.5
	Articular‐haematologic	6	9.7
	Articular‐pulmo‐cardiac	2	3.2
	Not specified	28	45.2

BMI	Normal	26	41.9
	Underweight	6	9.7
	Overweight	24	38.7
	Obesity	6	9.7

Renal manifestations	No	42	67.7
	Yes	20	32.3

Clinical presentation	Cutaneous‐mucosal involvement	20	32.3
	Articular pain	20	32.3
	Cardiovascular involvement	3	4.8
	Neuropsychiatric involvement	6	9.7
	Pleuro‐pulmonary involvement	7	11.3
	Sicca syndrome	6	9.7

Synthetic antimalarials	No	6	9.7
	Yes	46	74.2
	Not specified	10	16.1

Immunosuppressive agents	No	30	48.4
	Yes	12	19.4
	Not specified	20	32.3

Treatment outcome	Improved	32	51.6
	Unchanged	2	3.2
	Not specified	28	45.2

*Note: n* = 62.

**Table 2:** Laboratory parameters.

Parameters	Categories	*n*	%	Median	Min–Max
Proteinuria ≥0.5 g/24 h	No	39	62.9	—	—
	Yes	23	37.1	—	—

Hypercreatininemia	No	42	67.7	—	—
	Yes	20	32.3	—	—

Antiphospholipid antibody syndrome	No	56	90.3	—	—
	Yes	6	9.7	—	—

ANA	—	60	—	1185	2.8–1280
Anti‐dsDNA	—	58	—	77	8–600
Anti‐Sm	—	56	—	19.5	2–320
Anti‐U1 RNP	—	14	—	10	3–241
Anti‐SSA	—	44	—	71.5	1–640
Anti‐SSB	—	30	—	12	2–640

*Note: n* = 62.

### 3.2. Distribution of CRP Concentrations According to Socio‐Demographic Factors

The mean age was 35.45 ± 13.50 years, ranging from 18 to 67 years. The mean BMIwas 25.0 ± 5.19 kg/m^2^, ranging from 16.49 to 43.12 kg/m^2^. CRP had a mean concentration of 28.20 ± 21.21 mg/L (median: 23.93 mg/L, range: 0.89–98.36 mg/L).

According to stratification by age group, 74.2% of participants were aged 18–45 years and 25.8% were aged 46–67 years. Regarding occupation, 16.1% were unemployed, 17.7% were students, 11.3% were engaged in informal work and 54.8% were in formal employment. No significant association was observed between CRP categories and age, BMI or occupation (*p* > 0.05) (Table 3).

**Table 3:** Socio‐demographic Characteristics by CRP categories.

Socio‐demographic characteristics	CRP (mg/L)			Total	*P* value
	<5	5–20	>20		
	*n* (%)	*n* (%)	*n* (%)	*n* (%)	
Age groups (years)					
18–45	5 (8.1)	12 (19.4)	29 (46.8)	46 (74.2)	0.704
46–67	3 (4.8)	4 (6.5)	9 (14.5)	16 (25.8)	—
BMI (kg/m^2^)					
Normal	4 (6.5)	4 (6.5)	18 (29.0)	26 (41.9)	—
Underweight	1 (1.6)	1 (1.6)	4 (6.5)	6 (9.7)	0.382
Overweight	3 (4.8)	10 (16.1)	11 (17.7)	24 (38.7)	—
Obesity	0	1 (1.6)	5 (8.1)	6 (9.7)	—
Occupation					
Unemployed	2 (3.2)	2 (3.2)	6 (9.7)	10 (16.1)	—
Student	2 (3.2)	1 (1.6)	8 (12.9)	11 (17.7)	0.337
Informal employment	2 (3.2)	2 (3.2)	3 (4.8)	7 (11.3)	—
Formal employment	2 (3.2)	11 (17.7)	21 (33.9)	34 (54.8)	—

*Note:* Fisher –– Freeman–Halton exact test.

### 3.3. Distribution of CRP Concentrations According to Participants’ Medical History

Among the study population, 9.7% had a family history of autoimmune disease and 9.7% were smokers. Those with a family history of autoimmune disease all had CRP levels >20 mg/L, with no significant difference (*p* = 0.190). Similarly, no significant association was observed with smoking status (*p* = 0.144) or with the onset of a first lupus flare‐up (*p* = 0.421). Finally, the distribution of flare‐up types according to CRP levels did not differ significantly (*p* = 0.287), despite a predominance of joint‐involving forms in the CRP >20 mg/L category (Table 4).

**Table 4:** Participants’ medical history by CRP categories.

Medical history	CRP (mg/L)			Total	p value
	<5	5–20	>20		
	*n* (%)	*n* (%)	*n* (%)	*n* (%)	
Family history AID					
No	8 (12.9)	16 (22.6)	32 (51.6)	56 (90.3)	0.190
Yes	0	0	6 (9.7)	6 (9.7)	—
Smoking status					
No	6 (9.7)	14 (22.6)	36 (58.1)	56 (90.3)	0.144
Yes	2 (3.2)	2 (3.2)	2 (3.2)	6 (9.7)	—
First lupus flare					
No	8 (12.9)	14 (22.6)	30 (48.4)	52 (83.9)	0.421
Yes	0	2 (3.2)	8 (12.9)	10 (16.1)	—
Flare type					
Cutaneous	0	1 (2.9)	1 (2.9)	2 (5.9)	—
Pulmo‐cardiac	0	1 (2.9)	1 (2.9)	2 (5.9)	—
Articular	1 (2.9)	4 (11.8)	13 (38.2)	18 (52.9)	0.287
Articular‐cutaneous	2 (5.9)	0	2 (5.9)	4 (11.8)	—
Articular‐haematologic	2 (5.9)	1 (2.9)	3 (8.8)	6 (9.7)	—
Articular‐pulmo‐cardiac	1 (2.9)	0	1 (2.9)	2 (5.9)	—

*Note:* Fisher–Freeman–Halton exact test.

### 3.4. Distribution of CRP Concentrations According to Clinical Parameters

In this cohort, 84.5% presented with asthenia and 45.2% with arthralgia or arthritis. The pain assessment (VAS) showed that 45.2% of patients were pain‐free, 24.2% had mild pain, 17.7% had moderate pain and 12.9% had severe pain. A statistically significant association was observed with the presence of arthralgia/arthritis (*p* = 0.044), with a higher proportion of CRP >20 mg/L in symptomatic patients. Similarly, the level of pain assessed by the VAS was significantly associated with CRP categories (*p* = 0.023), with moderate to severe forms being more frequently found in the CRP >20 mg/L category. Renal manifestations were strongly associated with CRP levels (*p* = 0.001), with all affected patients having CRP >20 mg/L.

However, no significant association was found between CRP categories and other clinical parameters, notably asthenia (*p* = 0.382) or the various clinical presentations (*p* = 0.982) (Table 5).

**Table 5:** Clinical parameters by CRP categories.

Clinical parameters	CRP (mg/L)			Total	p value
	<5	5–20	>20		
	*n* (%)	*n* (%)	*n* (%)	*n* (%)	
Asthenia					
No	4 (6.5)	7 (11.3)	11 (17.7%)	22 (35.5)	0.382
Yes	4 (6.5)	9 (14.5)	27 (43.5%)	40 (84.5)	—
Arthralgia/arthritis					
No	4 (6.5)	13 (21.0)	17 (27.4%)	34 (54.8)	0.044
Yes	4 (6.5)	3 (4.8)	21 (33.9%)	28 (45.2)	—
VAS pain level	—	—	—	—	0.023
No pain	4 (6.5)	13 (21.0)	11 (17.7%)	28 (45.2)	0.002
Mild pain	1 (1.6)	2 (3.2)	12 (19.4%)	15 (24.2)	0.232
Moderate pain	2 (3.2)	1 (1.6)	8 (12.9%)	11 (17.7)	0.364
Severe pain	1 (1.6)	0	7 (11.3%)	8 (12.9)	0.183
Renal manifestations					
No	8 (12.9)	16 (25.8)	18 (29.0%)	42 (67.7)	0.001
Yes	0	0	20 (32.3%)	20 (32.3)	—
Clinical presentation					
Cutaneous‐mucosal involvement	2 (3.2)	6 (9.7)	12 (19.4%)	20 (32.3)	—
Articular pain	2 (3.2)	5 (8.1)	13 (21.0%)	20 (32.3)	—
Cardiovascular involvement	0	1 (1.6)	2 (3.2%)	3 (4.8)	0.982
Neuropsychiatric involvement	1 (1.6)	1 (1.6)	4 (6.5%)	6 (9.7)	—
Pleuro‐pulmonary involvement	1 (1.6)	2 (3.2)	4 (6.5%)	7 (11.3)	—
Sicca syndrome	2 (3.2)	1 (1.6)	3 (4.8%)	6 (9.7)	—

*Note:* Chi‐square test for renal manifestations, Fisher–Freeman–Halton exact test for others.

### 3.5. Distribution of CRP Levels According to Laboratory Parameters

Among the participants, the majority had a CRP level >20 mg/L, regardless of the laboratory parameter being considered. Nearly one‐third of patients (30.6%) with proteinuria ≥0.5 g/24 h had a CRP level >20 mg/L, with a statistically significant association (*p* = 0.037). The same was true for hypercreatininaemia, with a *p* value of 0.001. Leukocytosis, observed in 72.6% of patients, was also strongly associated with elevated CRP, with nearly 60% having a value >20 mg/L (*p* = 0.001). On the other hand, thrombocytopenia (16.1%), anaemia (64.5%), the Coombs test result and antiphospholipid syndrome showed no statistically significant association with CRP concentration (*p* > 0.05). Thus, this table highlights a significant association between elevated CRP and three laboratory parameters: proteinuria, hypercreatininaemia and leukocytosis (Table 6).

**Table 6:** Main biological parameters by CRP categories.

Biological parameters	CRP (mg/L)			Total	p value
	<5	5–20	>20		
	*n* (%)	*n* (%)	*n* (%)	*n* (%)	
Proteinuria ≥0.5 g/24 h					
No	7 (11.3)	13 (21.0)	19 (30.6)	39 (62.9)	0.037
Yes	1 (1.6)	3 (4.8)	19 (30.6)	23 (37.1)	—
Hypercreatininemia					
No	7 (11.3)	16 (25.8)	19 (30.6)	42 (67.7)	0.001
Yes	1 (1.6)	0	19 (30.6)	20 (32.3)	—
Thrombocytopenia					
No	8 (12.9)	14 (22.6)	30 (48.4)	52 (83.9)	0.415
Yes	0	2 (3.2)	8 (12.9)	10 (16.1)	—
Anaemia					
No	4 (6.5)	7 (11.3)	11 (17.7)	22 (35.5)	0.382
Yes	4 (6.5)	9 (14.5)	27 (43.5)	40 (64.5)	—
Leukocytosis					
No	6 (9.7)	10 (16.1)	1 (1.6)	17 (27.4)	0.001
Yes	2 (3.2)	6 (9.7)	37 (59.7)	45 (72.6)	—
Coombs test					
Negative	0	0	6 (9.7)	6 (9.7)	—
Not performed	7 (11.3)	12 (19.4)	19 (30.6)	38 (61.3)	0.184
Positive	1 (1.6)	4 (6.5)	13 (21.0)	18 (29.0)	—
Antiphospholipid antibody syndrome					
No	8 (12.9)	16 (25.8)	32 (51.6)	56 (90.3)	0.183
Yes	0	0	6 (9.7)	6 (9.7)	—

*Note:* Fisher–Freeman–Halton exact test.

### 3.6. Correlation Between CRP and the SLEDAI Score

Figure 1 shows a significant positive correlation between CRP and the SLEDAI score (*r* = 0.323; *p* = 0.012). The higher the disease activity score, the greater the tendency for CRP concentrations to increase.

**Figure 1:**
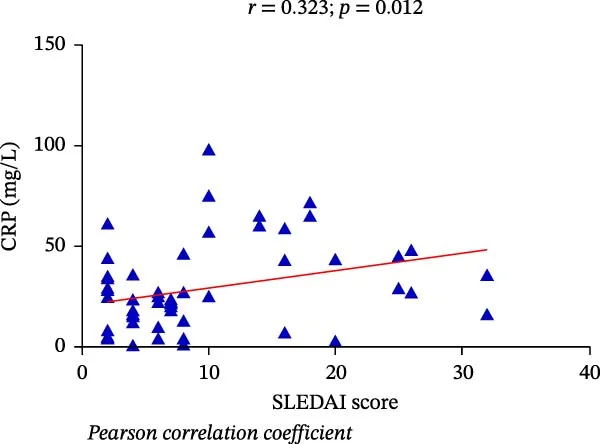
Correlation between serum CRP concentration and disease activity score (SLEDAI) in patients with systemic lupus erythematosus.

### 3.7. Binary Logistic Regression

The model included age, BMI, proteinuria ≥0.5 g/24h, hypercreatininaemia and SLEDAI. The table below presents the adjusted ORs, 95% CI, and *p* values. The (CI) were wide. After adjustment, hypercreatininaemia remained significantly associated with CRP ≥ 20 mg/L (adjusted OR = 24.8; *p* = 0.011), whereas the other variables showed no statistically significant associations (Table 7). The ROC curve (Figure 2) shows the probability predicted by the multivariate logistic model for identifying patients with CRP ≥ 20 mg/L. The AUC was 0.840. The curve deviated from the line of non‐discrimination, illustrating the distribution of sensitivity as a function of 1 –specificity for the different probability thresholds.

**Figure 2:**
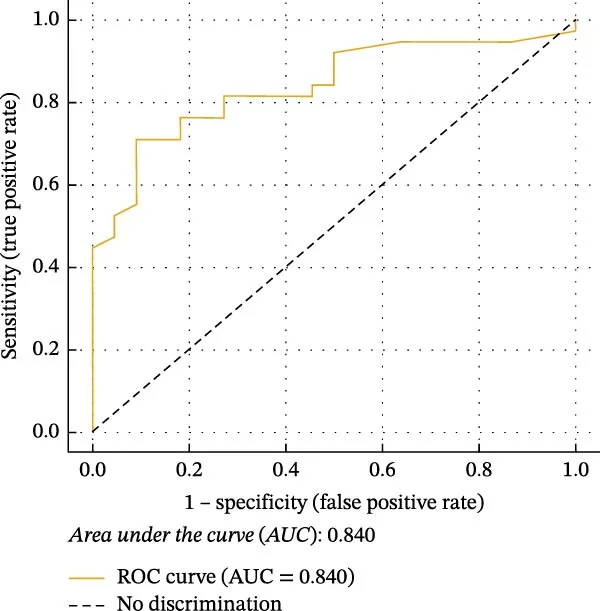
ROC curve for the predictive score of the logistic model for detecting CRP >20 mg/L.

**Table 7:** Multivariate logistic regression model of predictors for CRP ≥20 mg/L.

Variables	Wald	OR	95% CI	p value
Age (years)				
18–45	—	—	—	—
46–67	0.444	0.589	[0.124 – 2.797]	0.505
BMI				
Normal	4.281	—	—	0.233
Underweight	1.271	0.148	[0.005 – 4.107]	0.259
Overweight	0.417	0.611	[0.137 – 2.722]	0.518
Obesity	1.702	5.026	[0.444 – 56.849]	0.192
Hypercreatininemia				
No	—	—	—	—
Yes	6.506	24.796	[2.103 – 292.312]	0.011
Proteinuria ≥0.5 g/24 h				
No	—	—	—	—
Yes	0.755	2.080	[0.399 – 10.853]	0.385
SLEDAI	0.072	1.019	[0.889 – 1.168]	0.709
Constante	0.051	0.846	—	0.822

### 3.8. Estimation of Marginal Effects

The predicted probabilities showed an increased likelihood of elevated CRP associated with hypercreatininaemia (+47.5 points, *p* < 0.001) and proteinuria >0.5 g/24 h (+31.3 points, *p* < 0.001). With regard to BMI, being overweight was associated with a decrease (−19.2 points, *p* = 0.0155), whilst obesity was associated with an increase (+14.1 points, *p* = 0.0043). No significant effect was observed for the age group 46–67 years vs. 18–45 years (*p* = 0.293). Each + 1 point of SLEDAI increases on average the probability of elevated CRP of about 1.4 points (*p* = 0.0092) (Table 8).

**Table 8:** Average marginal effects calculated from the predictive score of the logistic model for detecting a CRP >20 mg/L.

Predictor	AME	SE	95% CI low	95% CI high	*Z*	p value
Age (years)						
46–67 vs 18–45	−0.0966	0.0933	−0.2771	0.0866	−1.0348	0.300
BMI						
Underweight vs. normal	−0.0256	0.1502	−0.3485	0.2314	−0.1707	0.864
Overweight vs. normal	−0.1923	0.0794	−0.3422	−0.0335	−2.421	0.015
Obesity vs. normal	0.141	0.0493	0.0433	0.2381	2.8578	0.004
Hypercreatininemia						
Yes vs No	0.475	0.0314	0.4165	0.5376	15.1203	<0.001
Proteinuria ≥0.5 g/24 h						
Yes vs. No	0.3126	0.0584	0.1907	0.421	5.3502	<0.001
SLEDAI score (per + 1)	0.0139	0.0053	0.0046	0.0252	2.6061	0.009

*Note:* Standard error (SE), 95% confidence intervals (95 % CIs), *z* statistic and *p* value.

## 4. Discussion

The results of this study highlight a frequent elevation in CRP levels among Ivorian female patients with SLE, with many CRP values ≥20 mg/L. Descriptive analysis, logistic regression and marginal effects estimation enabled the identification of the main clinical and biological determinants associated with this systemic inflammation. Hypercreatininaemia, significant proteinuria and obesity were major factors, highlighting the central role of renal involvement and metabolism in the biological expression of lupus activity.

In SLE, hepatic CRP production is generally inhibited by type I interferon [4, 22]. On the other hand, a marked increase in CRP, particularly above ~20–24 mg/L, should primarily raise suspicion of a concurrent infection, whilst bearing in mind that active serositis, arthritis or nephritis may also cause a marked elevation [4, 5, 23]. In our cohort, CRP correlated positively with the SLEDAI score, suggesting that it reflects, at least in part, the overall disease activity [5, 24]. The significant association with renal manifestations, arthralgia/arthritis and pain intensity reinforces this role.

Multivariate analysis confirms the importance of renal parameters in explaining systemic inflammation. Hypercreatininaemia proved to be the strongest predictor (OR 24.8; *p* = 0.011) and increased on average the probability of a CRP ≥ 20 mg/L of 47.5 points (*p* < 0.001). Proteinuria ≥0.5 g/24 h, although not significant in the adjusted model (OR = 2.08; *p* = 0.385), nevertheless increased this probability by 31.3 points (*p* < 0.001). In SLE, CRP may rise during active renal involvement, but its specificity is limited, hence; the, value of combined approaches (ESR/CRP ratio, PCT, NLR/PLR) to distinguish between infection and flare‐up [5, 23, 25]. The SLEDAI score was not significant in the regression analysis (OR = 1.02; *p* = 0.709) but its marginal effect was modest and positive (+1.4 probability points per unit, *p* = 0.007) [4, 22, 26].

In the study population, analysis of marginal effects revealed a differential effect of adiposity on CRP: being overweight reduced the likelihood of elevated CRP (–19.2 points; *p* = 0.015), whilst obesity increased it (+14.1 points; *p* = 0.004). This pattern is consistent with the literature indicating that an increase in BMI is associated with higher CRP concentrations, including in young adults aged 17–39 (indicating low‐grade systemic inflammation) [27], and that BMI is a major determinant of CRP levels from childhood onwards (the best predictor in a multiple regression analysis) [28]. Conversely, data on weight loss show that losing weight reduces CRP levels [29].

Finally, the ROC curve for the predictive score showed an area under the curve of 0.84, indicating excellent discriminatory performance, in line with the standards reported for clinical models applied to SLE [5].

These results highlight the importance of combining logistic regression with marginal effect estimation to identify the most relevant determinants of systemic inflammation in SLE, particularly the role of renal involvement and obesity.

Elevated CRP levels, particularly those of 20 mg/L or higher, should prompt an immediate consideration of infection. However, our study has shown that kidney disease and obesity may also be associated with elevated CRP levels. Therefore, in practice, the clinical context, proteinuria, creatinine levels and complete blood count should be taken into account for a more accurate interpretation.

This study has several limitations. Its cross‐sectional and single‐centre nature, as well as the relatively small sample size, limit the generalizability of the results and do not allow for the establishment of temporal or causal relationships.

The SLICC/ACR Damage Index (SDI) was not collected in a standardised manner when the database was compiled. The available information, particularly regarding the irreversibility and duration of organ damage, was insufficient to reliably reconstruct this score, thus making it impossible to assess the influence of cumulative organ damage on CRP concentrations.

Furthermore, only one clinical and laboratory assessment was considered for each patient, and CRP levels were not measured repeatedly over time. Consequently, changes in CRP levels before and after treatment could not be assessed. Therefore, the potential influence of treatment on CRP concentrations cannot be excluded.

Finally, certain factors likely to influence CRP levels, notably subclinical infection, nutritional status or other unmeasured inflammatory factors, could not be fully taken into account. Larger, multicenter, longitudinal studies incorporating a standardised assessment of the SDI and repeated CRP measurements would be required to confirm these results.

Despite these limitations, the rigour of the data collection, the use of a multivariate model and the estimation of marginal effects lend considerable strength to the conclusions, whilst highlighting the need for larger‐scale longitudinal studies.

## 5. Conclusion

In this study, elevated CRP levels were primarily associated with renal manifestations, notably proteinuria and elevated serum creatinine levels, as well as disease activity, as assessed by the SLEDAI. These results suggest that CRP could serve as a complementary biomarker in the assessment of patients with SLE, particularly in the presence of renal involvement.

However, given the cross‐sectional nature of the study and the absence of repeated CRP measurements, these results should be interpreted as associations and do not allow for an assessment of the influence of treatments on the progression. Larger‐scale longitudinal studies, incorporating repeated CRP measurements and a standardised assessment of the SDI, are necessary to confirm these results.

**Nomenclature**
ACR/EULAR: American College of Rheumatology/European Alliance of Associations for Rheumatology
AID: Autoimmune disease
AME: Average marginal effects
AUC: Area under the curve
BMI: Body mass inde
CRP: C‐reactive protein
ESR: Erythrocyte sedimentation rate
NLR: Neutrophil‐to‐lymphocyte ratio
OR: Odds ratio
PCT: Procalcitonin
PLR: Platelet‐to‐lymphocyte ratio
ROC: Receiver operating characteristic curve
SLE: Systemic lupus erythematosus
SIHIO‐TS: Société Ivoirienne d’Hématologie, Immunologie, Oncologie, et Transfusion Sanguine (Ivorian Society of Hematology, Immunology, Oncology and Blood Transfusion)
SLEDAI: Systemic lupus erythematosus disease activity index
VAS: Visual analogue scale for pain evaluation.

## Funding

The authors declare that no specific funding was received for this research.

## Disclosure

All authors read and approved the final manuscript.

## Conflicts of Interest

The authors declare no conflicts of interest.

## Data Availability

The data that support the findings of this study are available upon request from the corresponding author. The data are not publicly available due to privacy or ethical restrictions.
